# Supplementary material for: A conserved metabolic signature associated with response to fast-acting anti-malarial agents
Source: Microbiol Spectr. 2023 Oct 6;11(6):e03976-22. doi: 10.1128/spectrum.03976-22 (PMC10714989; doi:10.1128/spectrum.03976-22)
Supplement: Supplemental figures — Fig. S1 to S3. [file spectrum.03976-22-s0001.pdf]

Supplementary figures

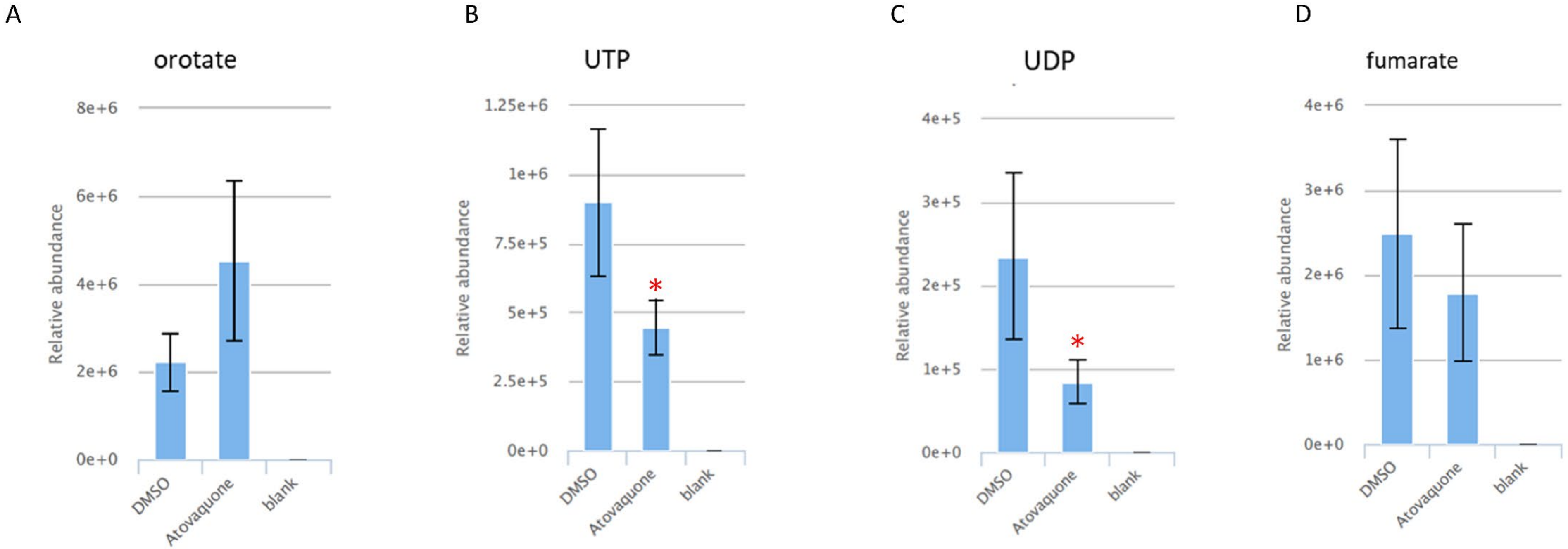

Fig. S1. Pyrimidine and TCA cycle pathway metabolite responses to ATQ treatment. Data was collected and processed as described in Fig. 2. Error bars are standard deviations. Significant changes are adjusted p-values of treatments as compared to DMSO. *P < 0.05.

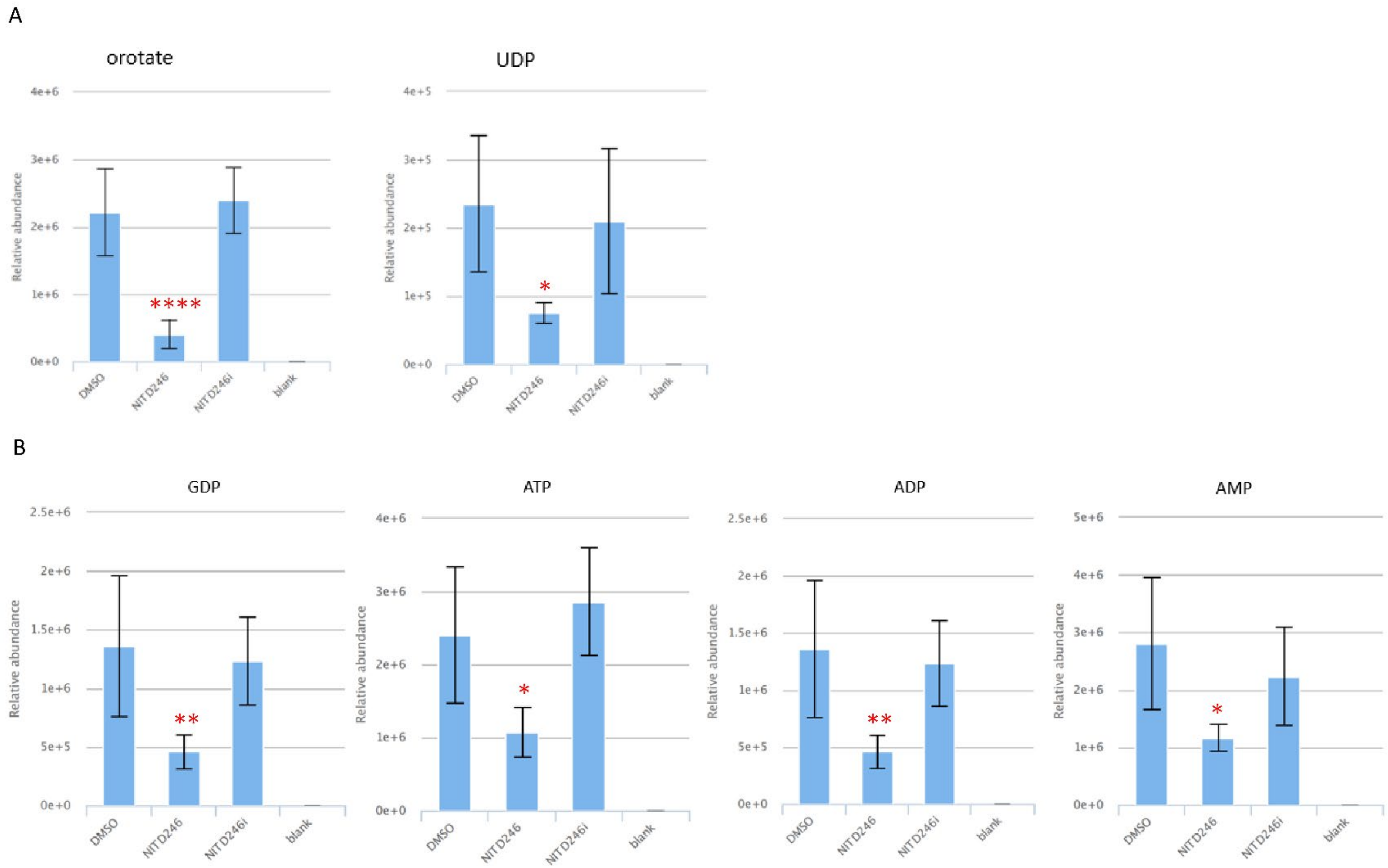

Fig. S2. **Relative abundance of indicated pyrimidine (A) and purine metabolites (B) metabolites in NITD246 treated parasites**. Data was collected and processed as described in Fig. 3. Error bars are standard deviations. Significant changes are adjusted p-values of treatments as compared to DMSO. *P < 0.05, **P < 0.01, **** P < 0.0001.

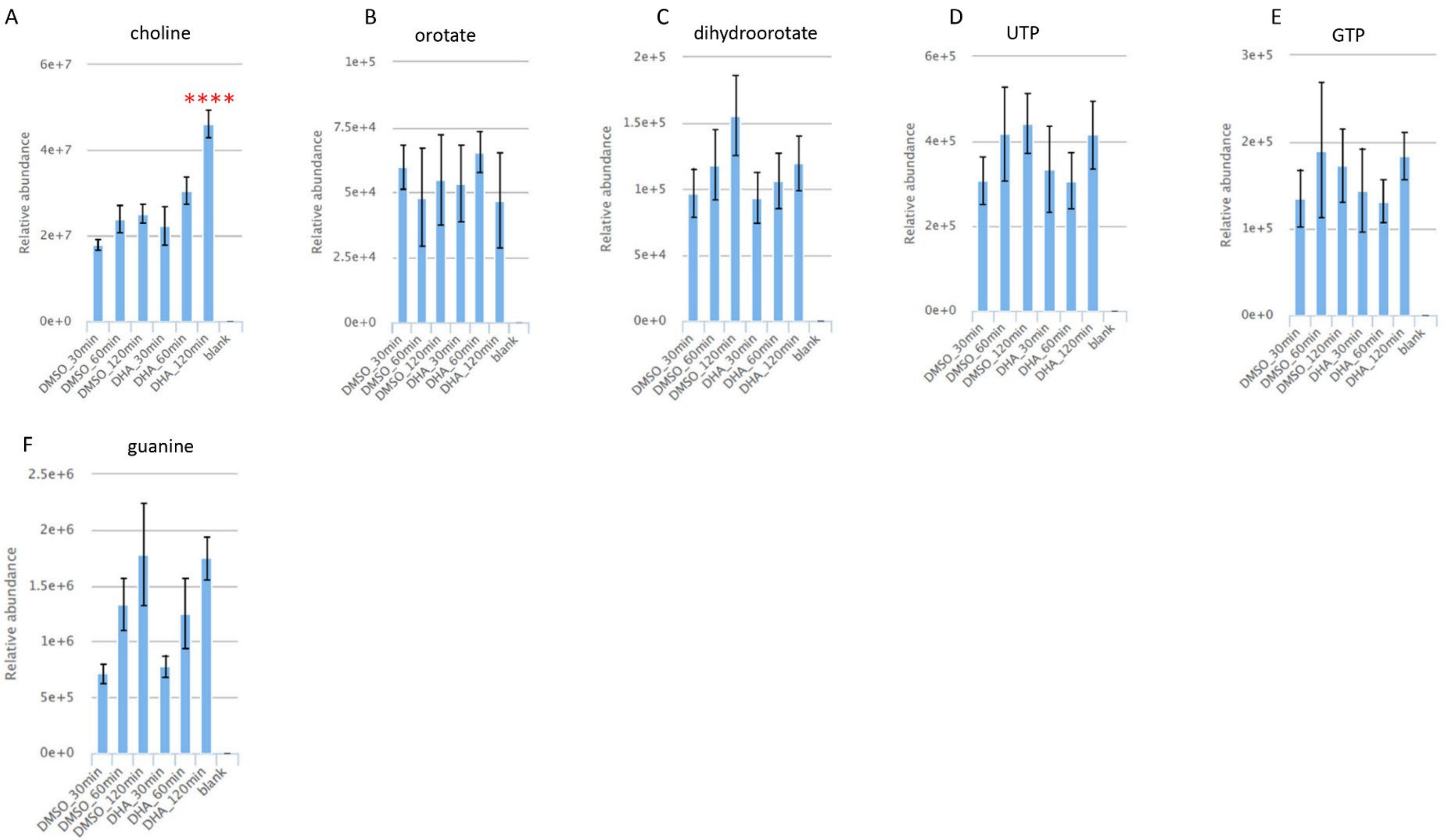

Fig. S3. DHA choline, purine and pyrimidine responses in malaria parasites exposed to the drug for 30, 60 or 120 minutes. Data was collected and processed as described in Fig. 7 for NITD246 and Cpd 55. Error bars are standard deviations. Significant changes are adjusted p-values of treatments as compared to DMSO for each individual time point. **** $P < 0.0001$.
